# Improved Spatiotemporal Framework for Human Activity Recognition in Smart Environment

**DOI:** 10.3390/s23010132

**Published:** 2022-12-23

**Authors:** Ziad Salem, Andreas Peter Weiss

**Affiliations:** Smart Connected Lighting Research Group, Institute for Surface Technologies and Photonics, Joanneum Research Forschungsgesellschaft mbH, Industriestrasse 6, 7423 Pinkafeld, Austria

**Keywords:** human activity recognition, inertial measurement unit, visible light sensing, sensor data fusion, features extraction, machine learning

## Abstract

The rapid development of microsystems technology with the availability of various machine learning algorithms facilitates human activity recognition (HAR) and localization by low-cost and low-complexity systems in various applications related to industry 4.0, healthcare, ambient assisted living as well as tracking and navigation tasks. Previous work, which provided a spatiotemporal framework for HAR by fusing sensor data generated from an inertial measurement unit (IMU) with data obtained by an RGB photodiode for visible light sensing (VLS), already demonstrated promising results for real-time HAR and room identification. Based on these results, we extended the system by applying feature extraction methods of the time and frequency domain to improve considerably the correct determination of common human activities in industrial scenarios in combination with room localization. This increases the correct detection of activities to over 90% accuracy. Furthermore, it is demonstrated that this solution is applicable to real-world operating conditions in ambient light.

## 1. Introduction

Electrical and optical microsystems belong among the key technologies of industry 4.0, Smart Home, Smart Building, Ambient Assisted Living technologies and Internet of Things (IoT) applications [1]. Inertial measurement sensors (IMUs), which include an accelerometer and a gyroscope and are often also combined with magnetometers, represent an important example for commercially very successful and often used microsystems, for instance in wearable devices and for physical activity monitoring [2]. During recent years, visible light sensing (VLS) and visible light positioning (VLP) methods have also received increased attention from researchers and are realized by integrated microsystems [3,4]. The task of VLS is to extract information from light received at photosensitive devices such as photodiodes (PD) or CMOS cameras and to use this information for various applications, such as occupancy detection, object identification, or gesture recognition. In VLP, objects equipped with reflective, light-emitting or light-receiving components can be localized through analyzing the light received by the photosensitive device by applying methods such as fingerprinting, proximity detection or geometric methods [5].

The aim of human activity recognition (HAR) is to recognize the physical activities of humans, such as daily routine actions such as walking and standing up/sitting down, and usually sensors or integrated microsystems are employed for this purpose. In industrial applications HAR is used, for example, to improve human-machine interaction or to provide application-related data for the user or for monitoring systems. Therefore, the development of advanced techniques to solve HAR problems is drawing more interest. One of these problems observed in current HAR activities is the disregard of location and time while monitoring the physical activity of persons. However, localization and location-based HAR systems significantly expand the range of application scenarios and are therefore also topics of the presented approach.

HAR systems are divided into vision-based (e.g., cameras) and sensor-based systems. Vision-based solutions have received more attention in recent years due to the introduction of RGB depth images; however, there are privacy and cost concerns with these systems [6]. Sensor-based devices including IMUs and other wearable devices allow for an interaction with the user and for collecting data in real time [7].

Besides the necessary hardware components for HAR solutions, machine-learning algorithms present a frequently used approach for data processing and prediction both for vision-based data and other sensor data. A common use case for wearable devices is to determine whether a person is currently consuming more or less energy (e.g., walking and running versus sitting) [8,9]. In this context, continuously performed basic activities such as walking and running can be detected with a single sensor, while detection in situations involving transitional activities such as sitting down on a chair, which are not repeated more frequently, is improved by using multiple sensor elements [10]. For the latter, sensor data fusion can thus provide a significant improvement in recognition accuracy.

Based on the combination of an IMU device and a RGB-sensitive PD it has already been demonstrated that activities such as walking versus standing are detected in different directions and rooms [11,12]. Sensor fusion thus provided a proof of principle for combining localization and HAR. This was achieved by using standard IMU devices, such as those used in smartphones, and implementing VLP without any changes to the existing lighting infrastructure. The advantage of the chosen approach was the easy and cost-effective implementation. Drawbacks regarding the recognition accuracy by the application of clustering algorithms in this previous work can be related to the usage of sensor raw-data and to the placement of sensor elements at improper parts of the body where the sensors readings are affected by noise and strong disturbances [13].

Therefore, the current work extends this approach by applying methods for feature extraction from the sensor raw-data. Features in the time and frequency domain are commonly used in dealing with HAR and for improving the overall accuracy of these systems [14,15]. Time-domain features range from mean, median, standards deviation, variance, minimum and maximum values, interquartile range, signal magnitude area (SMA), signal vector magnitude (SVM), to cross-correlation coefficients, only to mention some of the most important. Frequency domain features can include the power spectral density, power of short-time Fourier transform, autocorrelation coefficients, mean and median frequency, average of continuous wavelet transform or spectral entropy [16,17]. Time domain features are easily extracted from the raw data, whereas frequency domain features require higher computation and storage capacities [18]. Applications which already implement time and frequency domain features range from enhancing speech processing [19], image classification [20], HAR accuracy [8] to fall detection accuracy [21].

The scientific novelty of the paper includes the extension of basic human activities to additional complex activities based on feature extraction in the time and frequency domain. Furthermore, it combines human activity recognition with room localization for industrial applications under lighting conditions relevant for such application scenarios. Investigating in particular the applicability of our approach in the presence of ambient sunlight, it demonstrates real-world applicability where ambient sunlight cannot be ignored. Future research can build on and benefit from the insights found here.

This paper is organized as follows: Section 2 summarizes the current state-of-the art for time and frequency domain features extraction with HAR localization. Section 3 starts with an outline of the system for HAR and localization in industrial scenarios. Based on low-cost and low-power sensor elements, the proposed microsystem fuses IMU and VLP data, which are used as an input to feature extraction methods for signal processing and signal evaluation. The experimental conditions are outlined and details are given for the training as well as the online test procedures. Last but not least, the applied methods for feature extraction and data evaluation are presented. Section 4 shows the obtained results with a strong focus on real-world conditions with ambient light. Section 5 provides a conclusion and a few suggestions for future work.

## 2. Related Work

Advances in electrical and optical microsystems have also pushed HAR research activities in different fields. However, most of this work is based on IMU and especially accelerometer data, while positioning and localization are often not addressed. The latter can be related to the fact that for position determination commonly employed approaches are based on time of arrival (TOA), angel of arrival (AOA) or time difference of arrival (TDOA) in case of lower requirements concerning the position accuracy. Further, Direct Position Determination (DPD) [22], Kalman filter [23], Particle filter [24], and laser tracking measurements [25] are commonly used methods providing higher accuracy.

Yan et al. found that a combination of light detection systems with IMU data for mobile robot localization provide a higher precision compared to the individual sensor elements [26]. Ibrahim et al. investigated the determination and tracking of human activity through VLS technologies with sensor elements placed on the floor or worn by the person being studied [27]. Xu et al. proposed an indoor localization system using IMU and PD sensors on a smartphone [28], and Liang et al. applied VLP, IMU and a rolling shutter camera approach for improved position accuracy [29]. Hao et al. used a hybrid system with VLP for distance information and IMU for orientation [30]. Liang and Liu combined IMU motion data with camera measurements and LED markers for position estimation with high accuracy [31]. Hwang et al. presented results for a wrist-mounted device, which combined single camera RGB images with IMU data [32]. Finally, Poulose and Han proposed a hybrid indoor positioning system based on an IMU and a camera for minimizing positioning errors [33].

In addition to localization and activity recognition based on raw data processing and analysis, efforts to improve the performance were also made in the area of feature extraction methods and machine learning algorithms. Pires et al. considered walking, walking-downstairs, walking-upstairs, standing, running and sitting to be among the most frequently performed human activities [16]. Further, the authors summarized that mean, standard deviation, maximum, minimum, energy, interquartile range, variance, median and correlation coefficients belong among the most used features for extraction and that Artificial Neural Networks (ANN), Multi-Layer Perceptron (MLP), logistic regression, random forest and J48 are often used algorithms in HAR. Tian et al. extracted features such as mean, variance, minimum and maximum from accelerometer and gyroscope data [34]. This allowed determining several activities with high accuracy by using a SVM algorithm. Shen et al. also extracted several features from accelerometer and gyroscope data and determined human activities with SVM, KNN, MLP, and Random Forest algorithms [35]. Vallabh et al. applied a similar approach for the detection of people collapsing on the ground [36]. For this use case, the highest accuracy was found for SVM, Naïve Bayes, MLP and Least Squares Method (LSM) algorithms. Feature extraction from both accelerometer and gyroscope with J48, JRip, Random Forest, SVM, Naïve Bayes, MLP, Bagging, and KNN algorithms for HAR was demonstrated by Tang and Phoha [37]. Bulling et al. found varying results for the performance of HAR with accelerometer and gyroscope devices, depending on the number and the type of used features and in correlation to the sensor position and the type of sensor [38]. In general, accuracy depends on the amount of information extracted from a sensor dataset, such as room-level localization alongside HAR, and is usually countered by adding additional sensor elements. For example, a previous work demonstrated the recognition of different human activities together with room-level localization by combining data from multiple sensors [11].

For indoor localization and positioning, another approach is to use various radio frequency methods. These methods can provide accuracies of a few meters, but there are still limitations in terms of cost, complexity and the need for infrastructure modifications. Other methods, such as VLP, can provide high localization accuracy without the need for changes to the lighting infrastructure [39], depending on the solution approach. However, in most cases, this method is used for localizing a user in a room, while there is still a lack of research for room-level localization. Carrera et al. proposed a room-level localization system for person tracking based on a Wi-Fi fingerprint database created by smartphones in combination with magnetic field measurements [40]. With the help of Hidden Markov Models (HMM), the system demonstrated a high precision accuracy. Wojek et al. presented the tracking of several persons in their daily activities on a room level [41]. This was realized through gathering audio and video data features from camera and microphone systems placed in each room and employing a HMM algorithm for data processing and analysis.

Overall, given the state of the art just described, our work provides new insights into the application of HAR together with room-level localization. By combining different sensor technologies with feature extraction techniques, we investigate the performance of a system for industrial scenarios under realistic application conditions. The approach offers a low-cost and low-complexity solution without requiring any changes to the existing infrastructure.

## 3. Methods

The presented approach includes sensor data extraction, sensor data fusion and finally machine learning experiments. This section provides details about the proposed system, the training and the online test procedures, experimental conditions, and finally, also about data extraction.

### 3.1. System Description

Recorded datasets are composed of sensor data that are acquired on the one hand from an IMU device called Next Generation IMU (NGIMU) [42] that is used for human physical activity recognition (HAR), and on the other hand, from light data provided by an in-house designed VLP unit [11], which is used for the room detection. Data from the NGIMU includes accelerometer, gyroscope and magnetometer values.

The VLP receiver consists of a RGB sensitive PD, which provides three separate sensitive areas for the three different signal channels, Red (R), Green (G) and Blue (B), corresponding to three wavelength ranges in the visible light spectrum. In general, the PD delivers a current, which is proportional to the incident light in the respective spectral range. These current signals are separately detected and converted by Transimpedance Amplifiers (TIA) to voltage signals, which are connected to the built-in Analog-to-Digital Converter (ADC) of the NGIMU. The advantages of connecting the VLP unit with the internal ADC of the NGIMU are, first, that the provided communication interface is shared with the VLP sensor device and, second, that sensor readings of both devices, the NGIMU and the VLP unit, are synchronized and exhibit the same timestamp from the NGIMU.

Sensor signals are collected at a commonly used sampling rate of 100 Hz [43,44]. Further, the IMU device is connected to a laptop via Wi-Fi by an Open Sound Control (OSC) wireless communication protocol using a data transmission rate of 1 kHz. For the training sequences of the system (see Section 3.2) an additional hardware button is implement, which is pressed by the user to record the data relevant for the respective situation.

For having a clear line-of-sight (LOS) between light sources and the RGB PD sensor, the NGIMU device with its additional supporting circuit is placed on top of a helmet. Other works suggested placing the IMU on the front, on the back, or on the sides of the head as well as placing the sensors on the wrist, hip, foot, elbow, knee, or chest [45]. For the industrial application scenario envisioned here we consider the position on the helmet to give results with high accuracy while only minimally interfering with the user’s tasks.

**Figure 1:**
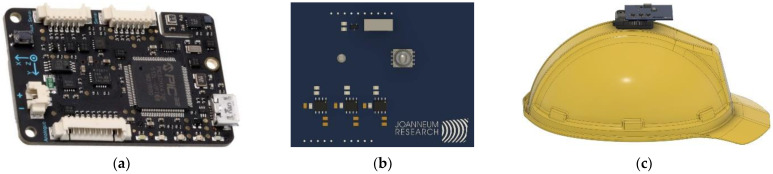
The hardware components used in this study: (**a**) NGIMU device; (**b**) VLP unit; (**c**) Side view of the helmet with integrated sensor devices and additional supporting circuit.

Figure 1a shows the NGIMU device with sensors placed in the top right corner [42]. Figure 1b depicts the in-house developed VLP unit with an RGB PD located middle-left and a LED on the middle-right side. The three TIAs can be seen on the bottom left corner. Figure 1c shows the side view of the helmet with the sensor equipment mounted on the top.

Overall, this work contributes to more detailed insights about human physical activity detection and room localization with a helmet-mounted sensor device. This is emphasized for comparing reported prediction accuracies in different works [46].

### 3.2. Training and Online Test Procedures

The open source machine-learning tool Weka [47] was applied for system training as well as for online test procedures. For the final evaluation of the results, a tenfold cross validation is used, where a recorded dataset is split in nine parts for training and one part for testing. Further, in-house developed Python scripts were employed for the extraction of time and frequency domain features, connecting the IMU device through Wi-Fi to a laptop, and for training as well as for online test procedures of the system.

**Figure A1:**
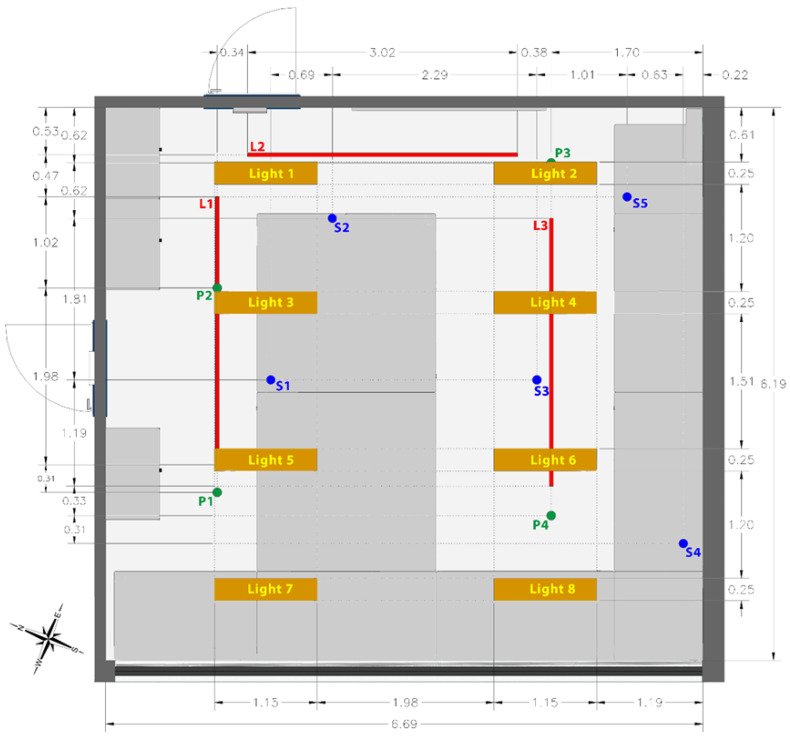
Room 1 (R1) floor plan with test points (P1–P4) and test lines (L1–L3) for the performance of physical activities and spectrum test points (S1–S5).

**Figure A2:**
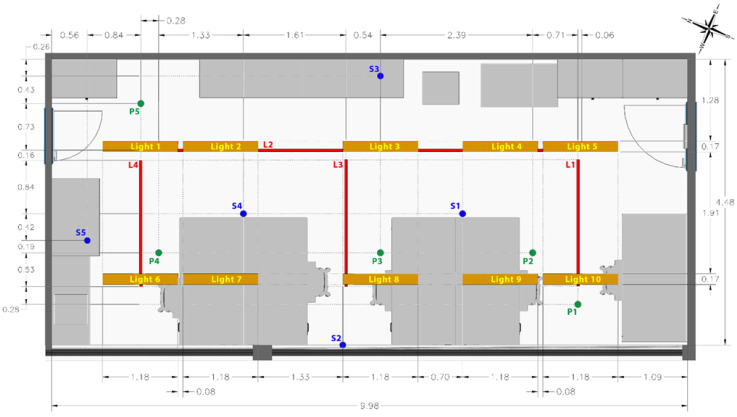
Room 2 (R2) floor plan with test points (P1–P5) and test lines (L1–L4) for the performance of physical activities and spectrum test points (S1–S5).

**Figure A3:**
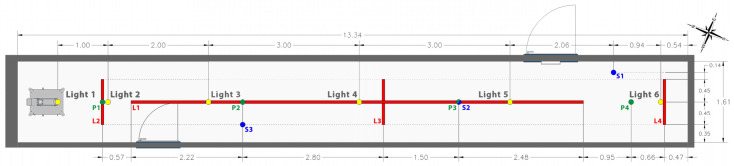
Room 3 (R3) floor plan with test points (P1–P4) and test lines (L1–L4) for the performance of physical activities and spectrum test points (S1–S3).

Training procedures for the VLP unit were performed with the room lighting on but blinds closed, i.e., without external ambient light. Data were recorded at all available points in each of the rooms (see Figure A1, Figure A2 and Figure A3 in the Appendix A). Further online tests were conducted under similar and under different lighting conditions, i.e., both closed and open blinds.

**Figure A4:**
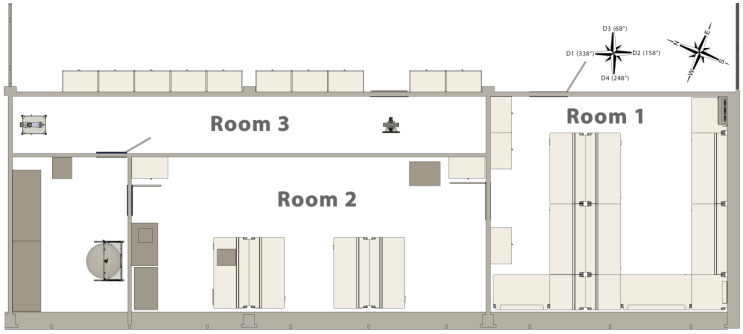
Top view of the three studied rooms with the definition of directions D1–D4.

For IMU sensor training, an arbitrary user collected data for a specific physical activity and assigned the respective activity label to this data. Physical activities included walk, no-walk (standing and sitting), sit-to-stand, and stand-to-sit in four defined directions of the rooms as specified in Figure A4. Subsequent feature extraction is based on measurements from the accelerometer sensor of the IMU. At the end of IMU sensor training, which in principle can be performed at any point in a room, the data from different activities are merged for further processing. However, the test series were planned so that the data were collected at different times, days and in different rooms. This was to provide the possibility to investigate whether these factors affect recognition accuracy. For example, Dillon et al. suggested that a wrist-worn accelerometer would need to be monitored for at least six days to reliably detect habitual activity [48].

Upon completion of the training procedures, the training dataset is processed using a common J48 algorithm to generate a data-driven decision tree, which is then converted into executable Python 3.9 code via a self-developed script. The generated Python code, which reflects the decision tree rules determined by the training data, is then used to evaluate the online tests.

A series of online tests was performed in all three rooms, R1, R2 and R3, for system performance evaluation. The online tests included walk, no-walk, sit-to-stand and stand-to-sit activities in the defined four directions D1 to D4 as shown in Figure A4. The walking activities were always carried out along set paths marked by lines on the floor and shown in red color in the room plans (“L”-characters, see Figure A1, Figure A2 and Figure A3). No-walk, sit-to-stand, and stand-to-sit activities were performed at test points marked on the floor and represented by green dots in the room plans (“P”-characters). The no-walk activity is represented by both stand-still and sit-still activities. Further, it should be emphasized that the activities studied here include both static and transitional human activities. Transitional activities are considered more complex for HAR, but they can provide a more complete activity description compared to using only static tasks. Thus, the presented approach allows an extended application under more realistic operational conditions.

The evaluation results of the online test series including room determination, activity determination and direction determination were stored for each experiment, and finally the number of correct determinations over all experiments was calculated. From this, the respective accuracy for correct predictions is derived.

### 3.3. Experimental Conditions

To explore the impact of variations in external light conditions during the day, a training procedure was conducted at a specific time and day, and the online tests were repeated at a later stage. Table 1 summarizes the parameters for the performed series of experiments, which were analyzed with the focus on room localization by the VLP unit.

To provide even more detailed information about the specific changes in light conditions during the experiments, the illumination level and the spectral light distribution were determined at the beginning of each experiment. The illumination (in units of lux, one lux is equal to one lumen per square meter [49]) was measured at various heights above the floor and at specified spectrum test points inside the three rooms. The spectrum test points are marked with “S”-characters in blue in the room plans—see Figure A1, Figure A2 and Figure A3—and visible light spectra were taken with an UPRtek Spectrometer MK350S Premium [50]. The respective heights of the spectrum test points from the floor are summarized in Table A1 in the Appendix. Illumination values and spectra are shown in the Section 4. By applying the training procedure described above, it is expected that the approach to HAR and room identification presented here can be applied to any indoor space under varying environmental and different lighting conditions, although the proposed system was only evaluated in three specific rooms in the experiments conducted here.

A second series of tests was carried out with regard to testing the accuracy for HAR. Table 2 gives an overview of this series of experiments. Data for five activities were collected on three different days and in two different rooms. The corresponding number of iterations and experiments for each of the activities is specified in this table, and the bottom line summarizes the merged data of all three previous days. The last column indicates how the data have been split for training and testing. An example of a captured data file can be found in the Section 3.4 (see Table 3).

Finally, it should be also mentioned that all experiments were conducted by a single person (male, age 55 years, 173 cm tall) wearing the helmet with the mounted sensor devices on his head. This is justified by the fact that, on the one hand, different people should not have a significant influence on the setup chosen here and, on the other hand, the major focus of the investigations is rather on different rooms and ambient conditions.

### 3.4. Data Extraction

For the purpose of this work, IMU and light data were extracted separately. Since the activity detection is location-independent, the experiments can in principle be performed at any location. In contrast, data acquisition for the VLP device must be conducted in the target rooms, since room identification is based on the specific lighting conditions of these rooms. Afterwards, IMU and light data from both sensor devices are fused to constitute a supervised data set for feature extraction in machine learning algorithms. Finally, the extracted features can be listed in single columns together with an additional column, which represents the class—for example, describing one of the physical activities.

**Figure 2:**
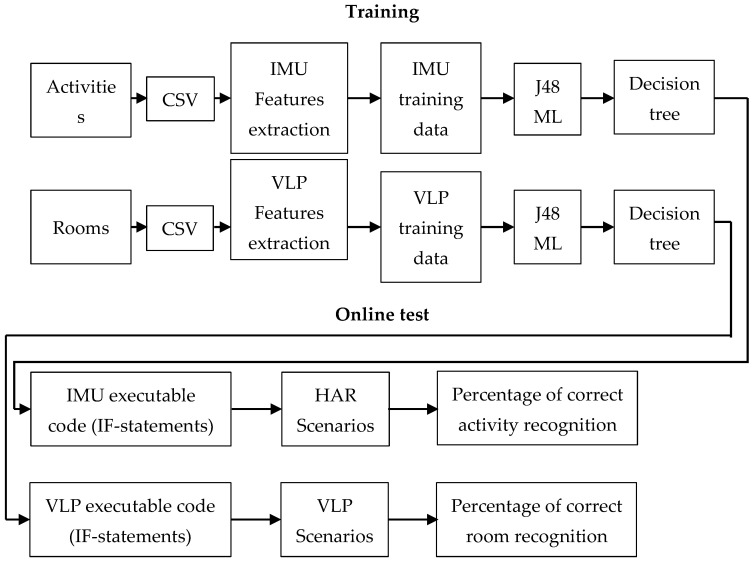
Our approach reference model.

Figure 2 shows the reference model of our approach and illustrates the processes of training and online testing.

#### 3.4.1. IMU Data

Accelerometer sensor data are the best suited to differentiate between various activities [51,52]. Therefore, IMU data is recorded, and an extraction of the time and frequency domain features applied to the measured values of acceleration in the three directions x, y and z is done. Data acquisition is performed for a duration of three seconds for each of the five different studied activities, and each individual activity is repeated several times and on different days. This results in a total number of 140 iterations for each activity. Table 3 shows an exemplary dataset generated from the accelerometer sensor data together with a timestamp and the classification for the respective data-related activity ADL (activity daily living).

For activity recognition in combination with room localization, the IMU data were acquired during all five considered activities. Further, all activities were performed in four directions of a room based on the room orientation as shown in Figure A4 in the Appendix (D1 to D4). A final data set representing all physical activities is formed by merging the data from all activities. In the next step, a set of time and frequency domain features is extracted; Table 4 shows the list of features as used in this study. These are features that are commonly employed in HAR approaches.

Table 5 shows an exemplary dataset for the extracted time and frequency domain features in relation to the physical activities ADL. For the sake of space and simplicity, this example only refers to *x*-axis accelerometer data.

Finally, for direction recognition, we used the quaternion message from the NGIMU, which describes the device’s orientation relative to the Earth [42]. The utilized NGIMU quaternion message is composed of four elements, namely the x, y, z and w information. The w value gives the rotation around the vectors x, y and z, which was consequently used for determining the direction of the system, as detailed in our previous work [11].

#### 3.4.2. Light Data

As stated in Section 3.1, the VLP unit generates three data channels, which are related to the detected light intensity in three regions of the visible light spectrum (RGB colors). The measurements of absolute values are more sensitive to noise compared to relative data characteristics based on, for example, ratios of raw data channels.

Due to the varying distance between the user and the light sources, the different illumination infrastructure in the rooms and the changing ambient light conditions, it is to be expected that the incident light at the PD sensor and thus the measured raw data are subject to significant variations. However, the spectral composition in a specific room is expected to be more reliable for data analysis, and therefore relative data are computed by either subtracting or dividing the sampled raw PD channel values. Based on these considerations, the following six features were generated: blue minus green (B-G), blue minus red (B-R), green minus red (G-R), blue divided by green (B2G), blue divided by red (B2R) and green divided by red (G2R).

Table 6 shows an example data set with the raw data values (B, G, R) measured by the VLP device and the subsequently calculated relative data characteristics.

## 4. Results and Discussions

In a first step, the data from the training and online tests 1 to 3 are evaluated and analyzed for recognition accuracy under changing ambient light conditions. In the second step, the focus is on the recognition accuracy with respect to the investigated activities for HAR. Finally, the influence of changing ambient light on room localization is discussed in more detail in Section 4.2.

### 4.1. Activity Recognition under Changed Ambient Light Conditions

Several options are usually available to evaluate the quality of systems like the presented approach, such as accuracy, precision, or recall. As with many other HAR systems, we apply the accuracy measurement, which specifies the average difference between correctly predicted and known values. The following equation for the ratio of correct predictions to the number of all predictions is applied to determine the accuracy:

accuracy = (TP + TN)/(TP + FP + TN + FN)(1)

True Positive (TP) refers to positive model predictions and the actual class being positive, True Negative (TN)—both, the model prediction and the actual class are negative. False Positive (FP) applies for a positive model prediction while the actual class is negative, and False Negative (FN) for negative model predictions with the actual class being positive.

**Figure 3:**
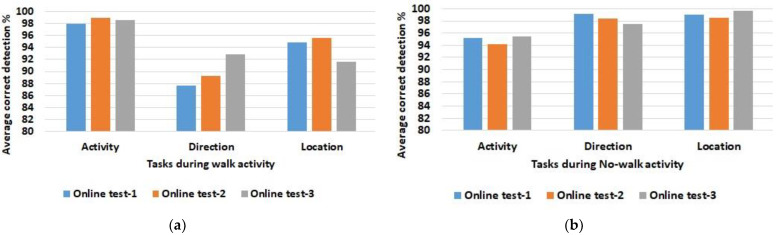
Averaged correct detections for activity, direction, and room localization for data from the experimental test series 1 to 3. Walk activity (**a**) at the left is compared to No-walk activity (**b**) at the right. Note that the *y*-axis ranges from 80–100% for better resolution of the differences between the results.

After a model has been created based on the training data, online tests 1 to 3 were evaluated in a first step. Since the focus is on room localization under varying ambient light conditions, only two activity classes are distinguished, namely walking and non-walking activities. Table 7 summarizes the averaged results for correct detections for walking activities during the online tests 1 to 3 and Table 8 shows the results corresponding to no-walk activities. This evaluation includes the detection of the performed activity, the direction of the activity, and the room localization. Further, Figure 3 visualizes the results.

First of all, it is clear from the results that the differences between the three online tests conducted for walking and non-walking activities are only slight. In more detail, the average of correct detections is a little bit higher for the no-walk activities. This is mainly attributed to increased noise for the walk activity as the sensor devices are in motion. Further, it must be mentioned that there are also locations inside the rooms which are not fully covered by the luminaires during the walk activity. Especially in room 3 (see Figure A3 in the Appendix) the distances between the light spots on the ceiling are not equal, so that light interruptions can occur between the sensor element and the light source, depending on the position of the person.

For further justification, the results of the online tests are compared taking into account the changing ambient light conditions. The difference between the average correct detections is calculated and summarized in the following Table 9 and Table 10. This comparison shows whether one of the online test series outperforms others in terms of correct detections.

This analysis also shows no major differences between changing conditions in the three online tests. Minor differences in detection accuracy for activity and direction when comparing walking and non-walking activities are most likely related to head movements and whether the helmet was properly fastened. Overall, these minor differences are considered small and negligible, and the system provides sufficient detection accuracy in combination with the selected features.

Keeping the focus on room localization under changing ambient light conditions, it can be stated that the three rooms are correctly detected with almost the same accuracy, regardless of whether the blinds of a room are open or closed. This is due to the fact that relative light features are used instead of the absolute raw data values acquired by the PD of the VLP unit. The results show that these relative features can avoid disturbances caused by changes in ambient light during the day, and furthermore, that these features are robust to intensity variations with changing distances between light sources and the PD sensor, especially during walking activity. Thus, the approach presented has been proven to provide a system with improved reliability for correct determinations.

In a second step, the focus is on HAR with a detailed analysis concerning the activities studied in the experimental tests. Further, the investigation focuses on the effects on detection accuracy that may be caused by the execution of individual activities at different places, days and times. Therefore, the recorded data are evaluated in two different ways:

- Experiment-1: evaluation includes 100 iterations measured on day 3 and site 2, i.e., this evaluation refers to data recorded on a single day and at a single location.
- Experiment-2: evaluation includes the sum of all 140 iterations (merged data) measured on three days and at two locations. Therefore, this evaluation refers to data comprising variations from different times and sites.

For these two experiments (evaluations), each data set is split into 70% of iterations for training and 30% for testing (also see the last column in Table 2), which is consistent with approaches often described in the literature.

The evaluation results are summarized in Table 11 and compared with several reported state-of-the-art attempts in this field. Differences between other studies and the presented results can be attributed to several issues, such as the number of samples used, the sampling rate, the activities chosen for HAR, the number and type of time and frequency domain features extracted, or the machine learning algorithm applied.

In general, the results in the literature show that in most of the studies, the time domain features perform better than the frequency domain features in almost every case. Meaning, the classification accuracy for features generated in the time domain is higher than that of those generated in the frequency domain. The results presented here show that the achieved accuracies beyond 90% can definitely compete with the results from other work. In combination with the extension of the system for direction detection and room localization, this demonstrates the strengths of the system.

A closer look at the results from experiment-1 and 2 shows that there is a minor decrease in the detection accuracy when comparing the results for data recorded on a single day and at a single location (96.67%) with data from three days and two different sites (91.43%). However, the achieved detection accuracy of over 90% is still considered as well tolerable.

For a better comparison of the activities executed on one day and on several days, a confusion matrix is calculated showing how many examples of each executed activity were correctly classified (diagonal values) and which were incorrectly classified by the algorithm [16].

Table 12 shows the results for the five activities when time domain features are applied in experiment-1 and experiment-2.

The correct classified activities are shown in the diagonal elements in the green boxes, incorrectly classified activities in the non-diagonal boxes in yellow. The percentages for misclassified activities in experiment-1 are 0.03% (St-si with Si-st), 0.03% (Walk with Si-st), and 1.02% (Stand with Sit), respectively. The percentages for misclassified activities in experiment-2 are 7.03% (Si-St with St-Si), 8.36% (St-Si with Si-St), and 5.60% (Sit with Walk), respectively.

Overall, it is assumed that the application of feature extraction methods in the time and frequency domains will provide sufficient recognition accuracy for the intended application scenarios.

### 4.2. Discussion of Different Ambient Light Conditions

To investigate the effects of changes in external ambient lighting conditions during the day, the illumination intensity and the spectral light distribution were measured at the beginning of each experiment (training and online tests). To further assess the differences between the three rooms and within each room, measurement points in the room at different heights above the floor were defined for these measurements (see Figure A1, Figure A2 and Figure A3 and Table A1 in the Appendix). Thus, different distances to windows and light sources are covered and included in the investigations.

**Figure 4:**
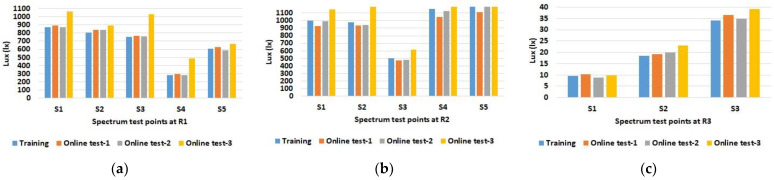
Determined illumination intensity (lux) for training and online test experiments at the defined spectrum test points S in room 1: (**a**) at the left with S1 to S5, room 2; (**b**) in the middle with S1 to S5, and room 3; (**c**) at the right with spectrum test points S1 to S3.

The results for the illumination intensities are given in Figure 4a–c. The values were determined for each of the defined spectrum test points in the three rooms.

**Figure 5:**
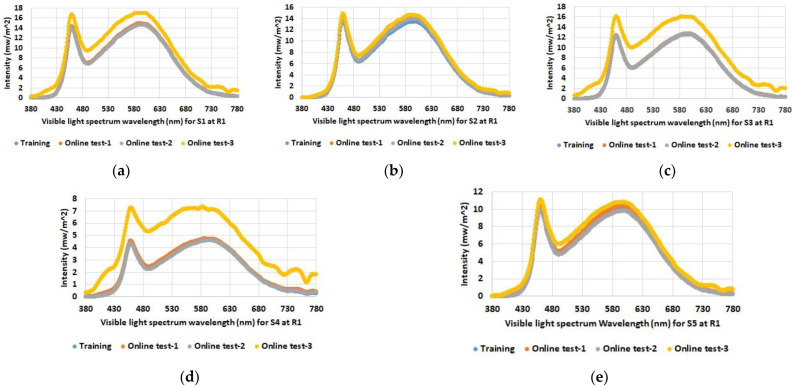
Determined spectral light distribution at defined spectrum test points in room 1: (**a**) S1; (**b**) S2; (**c**) S3; (**d**) S4; (**e**) S5.

**Figure 6:**
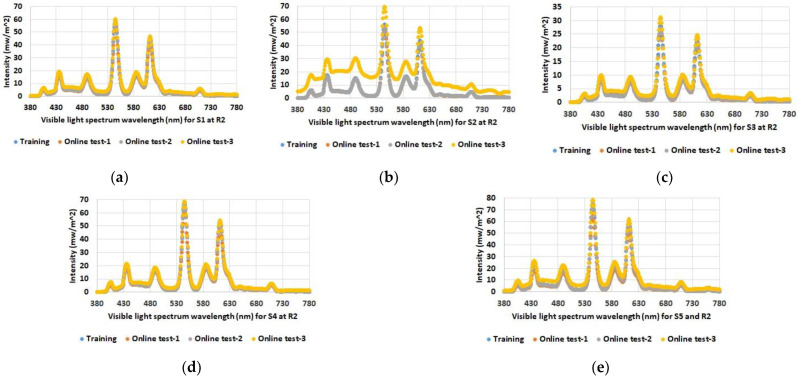
Determined spectral light distribution at defined spectrum test points in room 2: (**a**) S1; (**b**) S2; (**c**) S3; (**d**) S4; (**e**) S5.

**Figure 7:**
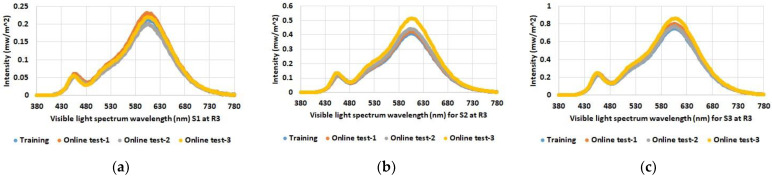
Determined spectral light distribution at defined spectrum test points in room 3: (**a**) S1; (**b**) S2; (**c**) S3.

As expected, the data show increased illuminance levels for spectrum test points near windows or indoor light sources. Furthermore, the increased illuminance for room 1 and 2 during online test 3 (yellow bars) with the blinds open and under sunny weather conditions (see Table 1) is also emphasized. The differences between the individual spectrum test points are also evident, such as between S2 and S3 in room 2, which are both at similar distances from the luminaires; however, the values for S2 near the windows are higher than the values for spectrum test point S3 on the wall opposite the windows. Overall, the figures also show that there were almost no differences between the training series and online test-1 and online test-2, although online test-2 was conducted with the blinds open but in cloudy weather conditions. The spectral light distribution was determined to be equal to the illumination intensity at the defined spectrum test points in the three rooms. The results for the comparison between the training and the online tests are shown in Figure 5, Figure 6 and Figure 7.

As with illumination intensity, the results for spectral distribution show that only online test-3 with open blinds and a clear sky has major differences from all other data. Moreover, this applies only to rooms 1 and 2, but as expected not to room 3, which has no windows. All other measurements show quite comparable spectra for training and online tests.

So far, it has been shown that the proposed system provides reasonable detection accuracy both when the blinds are closed and when they are open, and further when the blinds are open under both cloudy and sunny weather conditions. However, what has not been considered so far is sunlight directly entering the room. This can significantly alter conditions, as the illumination intensities are several magnitudes larger compared to a cloudy sky or to indoor illumination intensities. In addition, depending on the materials present in a room, there may be reflections of the incident sunlight that, when they impinge the PD sensing element of the VLP device, cause saturation of the signal. The saturation makes signal evaluation impossible; however, even with less severe impairments, the spectral distribution may be significantly altered at certain points in the room. Since the latter has a significant impact on the extracted features, it is to be expected that in these cases the detection accuracy will also be significantly impaired. Therefore, further investigations were carried out.

An additional online test was performed with the interior lighting switched on, the blinds open, and—this is new—sunlight shining directly into room 1 (spreading over an area of approximately 2.5 m from the windows into the room). For this test, a walking activity was chosen in which the test person walked along the defined path L3, which describes a straight path below the indoor lighting with varying perpendicular distances to the window (see Figure A1 in the Appendix). Table 13 shows the evaluation results for this experiment.

**Figure 8:**
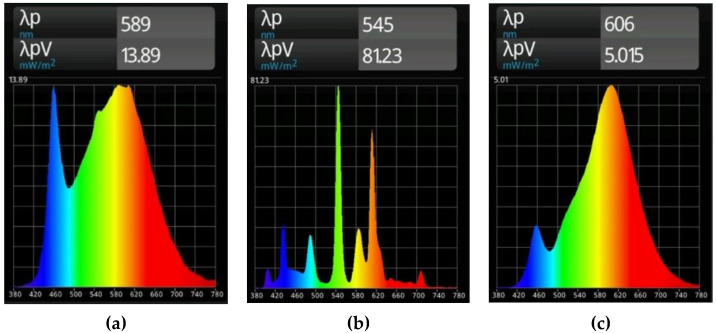
Comparison of spectral light distributions for the luminaires installed in room 1 (**a**); room 2 (**b**); and in room 3 (**c**). The *x*-axis represents the wavelength values in nanometers.

The results in Table 13 show that a person walks on the path L3 from the window towards the wall (direction D3—100%). However, in about the first half of the experiment (time ~1–5 s), instead of room 1, room 3 was incorrectly detected (R3—100%), which is attributed to the interference from the direct sunlight. Only in the second half of the experiment (time ~5–9 s), which refers to the part of L3 being closer to the wall and more distant from the window, does the algorithm correctly detect room 1 (R1—100%), as there is no interference from direct sunlight in this area. To support this interpretation, additional measurements regarding the illumination intensity and spectral distribution were performed. Figure 8a–c show the visible light spectrum of the three rooms, R1, R2 and R3. These measurements were taken directly under one luminaire in a room and at a height of 105 cm above the floor. The blinds of the room were closed and the interior lighting was on. Thus, the determined spectra refer to the characteristic properties of the lighting in each room.

The differences between the spectra of the three rooms are explained by the installed lighting systems, which were not modified in any way for the experiments performed. Room 1 is equipped with Osram LuxiLED 1200 × 300, 4000 K, 32 W luminaires, room 2 is equipped with Osram Lumiluix cool white FQ 54 840 W fluorescent lighting tubes, and room 3 is equipped with Philips CorePro LED Spots 4.6 W.

**Figure 9:**
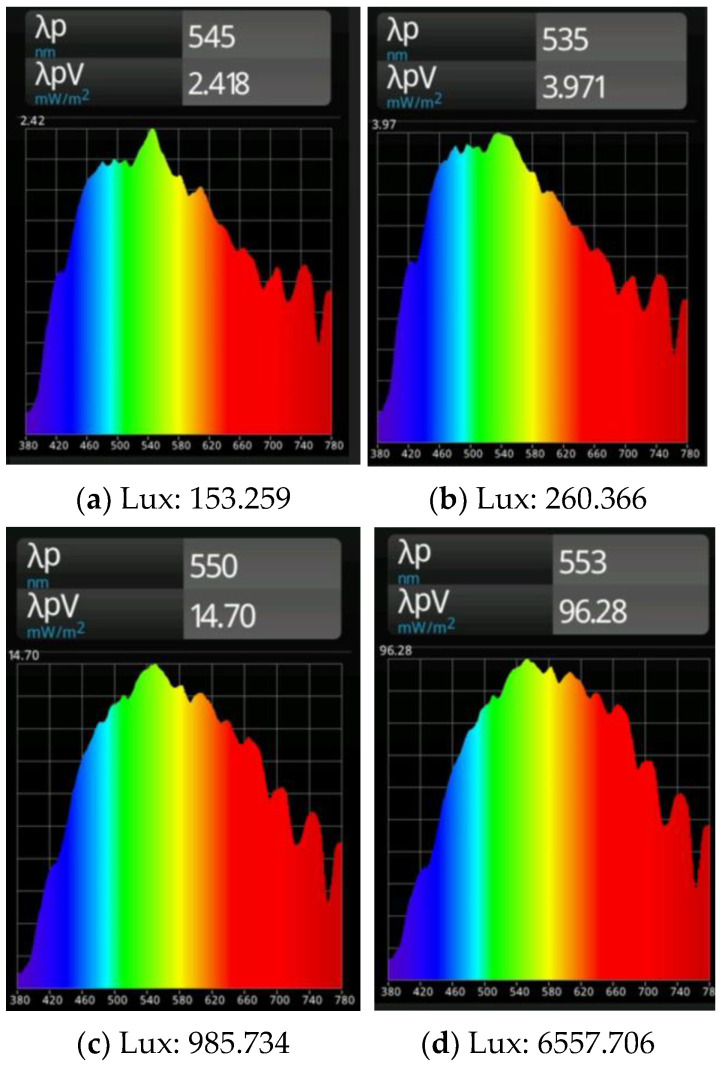
Comparison of illumination intensity (values below the figures in lux) and spectral distribution for the spectrum test points S1 located near the center of room 1 (**left**) and room 2 (**right**). Results at the top refer to foggy weather conditions (**a**,**b**); results in the bottom line to a partly cloudy sky (**c**,**d**).

Next, illumination intensities and spectral distributions were measured at points located more or less in the center of the rooms R1 (S1) and R2 (S1). The blinds were open and the interior lights were off to collect data related to the external ambient light conditions on a foggy day and under a partly cloudy sky. Figure 9 shows the corresponding results; λp refers to the peak wavelength within the spectral distribution, and λpV refers to the irradiance at the peak wavelength [50]. There are only minor differences in the spectral distributions but significantly increased illumination intensities for a partly cloudy sky compared to foggy weather conditions.

From these data, it can be inferred that the spectral distribution in the case of the dominance of sunlight from outside room 1 is more similar to the distribution in Figure 9c (sunlight spectra from outside of room 1) than to the distribution in Figure 8a (characteristic distribution for room 1). In addition, the distribution in Figure 9c appears to be more similar to the characteristic spectrum for room 3 (Figure 8c), which does not reflect the characteristic peak in the blue range of the room 1 illumination. Thus, the misclassification in certain areas of the experiment—identifying room 3 instead of room 1—can be explained by direct sunlight from outside interfering with the evaluation algorithm.

**Figure 10:**
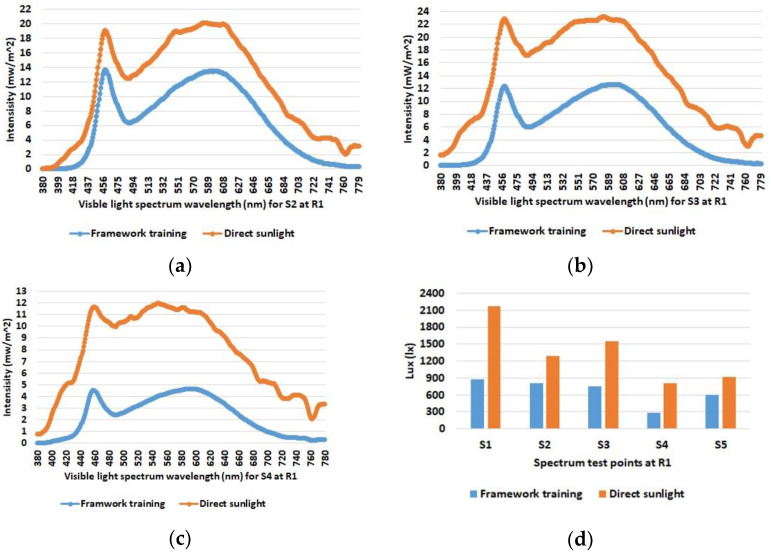
Spectral light distribution for the spectrum test points S2 (**a**); S3 (**b**); and S4 (**c**)—representing a diagonal line from the wall towards the windows in room 1 in comparison with (orange lines) and without (blue lines) direct sunlight. The illumination intensity at the five points in room 1 is compared in the given bar chart (**d**).

Figure 10 shows more measurements that show the influence of direct sunlight on the illumination intensity and the spectral distribution in room 1. The measurements were taken with the blinds open and the interior lighting switched on at a time when direct sunlight was shining into room 1. The comparison with the previous results without direct sunlight shows a clear increase in light intensities, which is also more pronounced at the measurement points near the window than at those near the wall. Furthermore, the characteristic peak of the room illumination in the blue frequency range also disappears more and more in the background of the solar spectrum.

Last but not least, the influence of temperature fluctuations, which are to be expected especially after switching on the lighting, when the light sources start to warm up, was investigated. This effect was found to have a negligible influence and the corresponding results can be found in Appendix B.

In summary, the presented approach is able to correctly identify different rooms under different conditions such as open or closed blinds as well as under different external weather conditions; however, it is compromised when direct sunlight interferes with the evaluation.

## 5. Conclusions and Outlook for Future Work

Monitoring human physical activities allows sophisticated applications in industrial scenarios. In addition, indoor localization and positioning is gaining high attention in recent years due to the wide availability of IoT applications in smart environments. A microsystem containing an NGIMU device and an in-house developed VLP unit was evaluated for HAR in combination with room identification for industrial applications. Several series of online tests were conducted for various physical activities, including basic and transitional activities ranging between sitting and standing, which are considered more difficult to detect with sensors. The results of this work were compared with a few previously published works in the field. Implementing time and frequency domain features in the evaluation algorithm demonstrated a stable performance of the approach for different environmental conditions in the three different investigated rooms. The results also show that the signal evaluation is only affected by interference from direct sunlight. Overall, the investigated system is considered to provide sufficient activities and room detection accuracies for various targeted industrial application scenarios.

Based on the achieved results, it is planned to extend the current presented approach for activity recognition and room identification by localizing a person within the respective rooms. In view of the application scenarios in the field of Ambient Assisted Living, activities such as lying down on a bed or lying on the floor will be also investigated. This needs to be accompanied by studying different positions for the placement of sensor devices on a person. Finally, additional evaluation metrics can be tested to increase detection accuracy, and the effects of having activities performed by different people also will be investigated.

## Figures and Tables

**Table 1:** Parameters and environmental conditions for the performed experiments.

Experiments	Indoor Lighting	Blinds	Start Time of Experiment	Weather Conditions
Training	ON	Closed	Day 1, 10:00	Cloudy
Online test-1	ON	Closed	Day 1, 13:00	Cloudy
Online test-2	ON	Opened	Day 1, 16:00	Cloudy
Online test-3	ON	Opened	Day 2, 09:00	Sunny, but no direct sunlight

**Table 2:** Description of the second experimental test series.

Experiments	Iterations	Total no. of Iterations	No. Measurements per Activity	Total no. of Measurements	Training vs. Test Samples
Day 1, Location#1	10	50	Sit: 2723 Si-st: 1716 Stand: 2628 St-si 2021 Walk: 2689	11,777	n.a.
Day 2, Location#1	30	150	Sit: 11410 Si-st: 7114 Stand: 11648 St-si: 7654 Walk: 9847	47,673	n.a.
Day 3, Location#2	100	500	Sit: 38580 Si-st: 24199 Stand: 32954 St-si: 21645 Walk: 33670	151,048	350 vs. 150
Merged data from three days and two locations	140	700	Sit: 52713 Si-st: 33029 Stand: 47230 St-si: 31320 Walk: 46205	210,497	490 vs. 210

**Table 3:** Example dataset for data generated from the 3-axis accelerometer sensor element (x, y and z-values in columns 2 to 4, respectively) with corresponding timestamp in the first column and activity classification in the last column (ADL—activity daily living. Five different activities are studied: stand—standing, sit—sitting, walk—walking, si-st—sit-to-stand and st-si—stand-to-sit).

Time (s)	Acce-X (m/s^2^)	Acce-Y (m/s^2^)	Acce-Z (m/s^2^)	ADL
0.1	0.161799952	0.017999846	0.981487095	sit
0.2	0.164758027	0.023350080	0.977752686	sit
0.3	0.170689359	0.034476221	0.982251704	sit
0.4	0.112286702	0.039446317	1.005190372	si-st
0.5	0.123579949	0.051039595	1.009228349	si-st
0.6	0.128493294	0.057355978	1.005034924	si-st
0.7	0.331447363	0.039430257	0.934785128	stand
0.8	0.331414938	0.035566427	0.930415988	stand
0.9	0.332439810	0.041852437	0.935305059	stand
1.0	0.170633137	0.026200492	0.985943556	st-si
1.1	0.169641167	0.023775911	0.985902011	st-si
1.2	0.168163225	0.021850912	0.983475626	st-si
1.3	0.293405503	−0.028735712	0.900109053	walk
1.4	0.302678615	−0.026843587	0.903027654	walk
1.5	0.310985833	−0.023983495	0.907395661	walk

**Table 4:** Time and frequency domain features used in this study.

Domain	Features	Description
Time	Mean	average based on the sum of the values divided by the number of values
	Median	middle number in a sorted list of numbers
	SD	Standard deviation: average amount of variability in the data
	IQR	Interquartile range: measure for the spread of the data
	Min	the lowest value
	Max	the highest value
	SVM	Signal vector magnitude: distinguishes between periods of activity and no-activity to identify when a person is doing an activity or not [45]
	SMA	Signal magnitude area: calculates the intensity of movement, which is important for detecting a fall [53]
Frequency	PSD	Power spectral density: a measure of the signal’s power. It shows at which frequencies the variation is strong or weak
	DF	Dominant frequency: refers to the component with highest sinusoidal magnitude [54]

**Table 5:** Example for a dataset that contains the extracted time and frequency domain features for various activities ADL (activity daily living).

ADL	Mean	Median	SD	IQR	Min	Max	SMA	DF	PSD
si-st	0.0270	0.0112	0.2635	0.4180	−0.418	0.5393	0.0270	4.0957	0.4166
si-st	0.1006	0.0410	0.2693	0.4717	−0.350	0.6080	0.1006	4.0260	0.5076
si-st	0.0657	−0.0195	0.2679	0.4451	−0.336	0.6621	0.0657	3.9124	0.4950
st-si	0.1259	0.16115	0.2816	0.5474	−0.348	0.5705	0.1259	4.0132	0.5882
st-si	0.0460	0.05314	0.2990	0.5293	−0.450	0.5425	0.0460	4.3323	0.4854
st-si	0.0280	0.04247	0.1315	0.5870	−0.484	0.4529	0.0280	4.3826	0.5209
sit	−0.1633	−0.1622	0.0142	0.0208	−0.194	−0.133	−0.163	2.6134	0
sit	−0.1503	−0.2150	0.0096	0.1013	−0.175	−0.127	−0.150	2.6521	0
sit	−0.1326	−0.1320	0.0116	0.0161	−0.155	−0.098	−0.132	2.2441	0
stand	−0.3144	−0.3153	0.0139	0.0217	−0.346	−0.271	−0.314	4.7428	0
stand	−0.2801	−0.2817	0.0179	0.0246	−0.313	−0.222	−0.280	4.5632	0
stand	−0.3228	−0.3226	0.0121	0.0148	−0.367	−0.294	−0.322	5.2891	0
walk	−0.2271	−0.2524	0.1219	0.1396	−0.471	0.1130	−0.227	4.0336	0
walk	−0.2835	−0.2963	0.1268	0.1864	−0.546	0.1038	−0.283	4.0899	0
walk	−0.3027	−0.3289	0.1239	0.1752	−0.555	0.0192	−0.302	5.2423	0

**Table 6:** Example for timestamped raw data acquired from the VLP unit and further extracted relative data in relation to the three different rooms R1 to R3 (Class).

Time	B	G	R	B2G	B2R	G2R	B-G	B-R	G-R	Class
0.1	161.66	145.61	152.88	111.01	105.74	95.247	16.044	8.7792	−7.265	R1
0.2	176.49	164.68	167.10	107.16	105.61	98.550	11.80	9.3847	−2.418	R1
0.3	177.40	155.90	157.11	113.78	112.90	99.229	21.494	20.283	−1.210	R1
0.6	139.56	104.14	107.77	134.01	129.49	96.629	35.419	31.797	−3.632	R2
0.7	141.98	103.23	103.83	137.53	136.73	99.416	38.749	38.144	−0.605	R2
0.8	145.31	98.691	102.02	147.23	142.43	96.735	46.621	43.291	−3.330	R2
1.1	209.18	207.37	205.55	100.87	101.76	100.88	1.8163	3.6328	1.8164	R3
1.2	209.18	207.07	205.85	101.02	101.61	100.58	2.1191	3.3300	1.2109	R3
1.3	209.18	207.37	205.85	100.85	101.61	100.73	1.8163	3.3300	1.5136	R3

**Table 7:** Average of correct detections for each task during walk activity based on data from all three online test series.

Correct Detection Average during Walk Activity (%)			
Experiments	Activity	Direction	Location
Online test-1	97.90	87.61	94.79
Online test-2	98.91	89.29	95.59
Online test-3	98.54	92.83	91.62

**Table 8:** Average of correct detections for each task during no-walk activity based on data from all three online test series.

Correct Detection Average during No-Walk Activity (%)			
Experiments	Activity	Direction	Location
Online test-1	95.25	99.20	98.99
Online test-2	94.12	98.43	98.58
Online test-3	95.49	97.47	99.61

**Table 9:** Differences between average correct detections in the three online tests for walk activity.

Average Detection Difference between the Online Tests for Walk Activity (%)			
Between	Activity	Direction	Location
Online test-1 and 2	1.01	1.68	0.80
Online test-1 and 3	0.64	5.22	−3.16
Online test-2 and 3	−1.37	3.54	−3.96

**Table 10:** Differences between average correct detections in the three online tests for no-walk activity.

Average Detection Difference between the Online Tests for No-Walk activity (%)			
Between	Activity	Direction	Location
Online test-1 and 2	−1.12	−0.76	−0.41
Online test-1 and 3	0.24	−1.73	0.62
Online test-2 and 3	1.37	−0.97	1.03

**Table 11:** Average detection accuracy (%) for two evaluations of experimental data (Exp-1 and Exp-2) in comparison with reported results in the literature. The accuracies are given for the combination of time and frequency domain features and also for time and frequency separately.

Domain	Exp-1	Exp-2	Study-1 [55]	Study-2 [16]	Study-3 [56]	Study-4 [57]	Study-5 [58]
Time & frequency	**96.67**	91.43	-	90.89	-	-	-
Time	96.67	91.43	93.52	-	87.00	**96.75**	95.00
Frequency	62.67	94.35	**94.34**	-	94.00	-	92.70

**Table 12:** Calculated confusion matrix for experiment-1 (left) and experiment-2 (right) when time domain features are applied.

Exp.-1							Exp.-2					
	Si-st	St-si	Walk	Sit	Stand			Si-st	St-si	Walk	Sit	Stand
**Si-st**	30	0	0	0	0		**Si-st**	35	7	0	0	0
**St-si**	1	29	0	0	0		**St-si**	6	36	0	0	0
**Walk**	1	0	29	0	0		**Walk**	0	0	42	0	0
**Sit**	0	0	0	28	0		**Sit**	0	0	5	37	0
**Stand**	0	0	**0**	1	29		**Stand**	0	0	0	0	42

**Table 13:** Results for an additional experiment with walking activity in room 1 and direct sunlight.

Time	R1	R3	D3	D4	Walk	No-walk	Rooms	ADL
1	0	100	0	0	100	0	R1	Walk
2	0	100	100	0	66.66	33.33	R1	Walk
3	0	100	100	0	85.71	14.29	R1	Walk
4	0	100	100	0	100	0	R1	Walk
5	85.71	14.29	100	0	83.33	16.67	R1	Walk
6	100	0	100	0	100	0	R1	Walk
7	100	0	100	0	100	0	R1	Walk
8	100	0	100	0	100	0	R1	Walk
9	100	0	100	0	100	0	R1	Walk
Ave.	53.97	46.03	98.14	0	92.86	7.14	-	-

**Figure A5:**
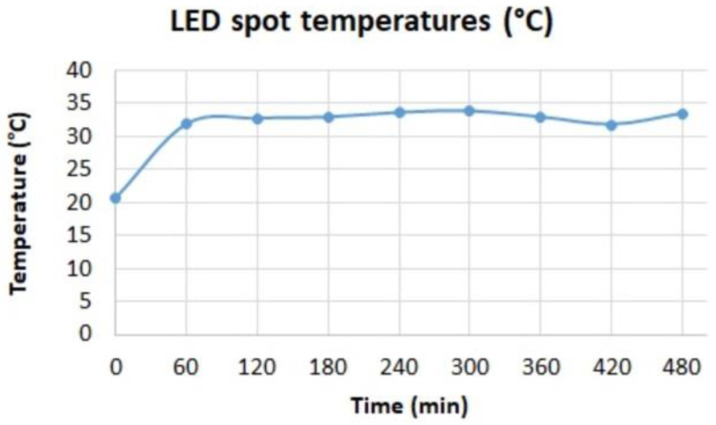
Measured temperature profile at one of the LEDs in room 3 after the LED was switched on.

**Figure A6:**
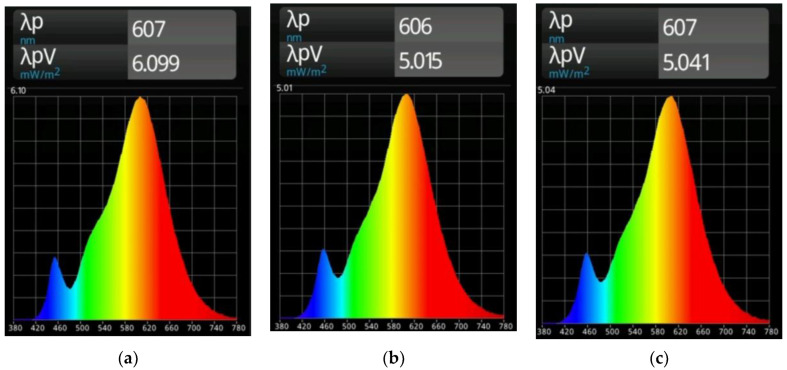
Comparison of spectral light distributions after switching on a LED in room 3. Measurements were taken at 0 min (**a**); 30 min (**b**), and at 60 min (**c**).

## Data Availability

Not applicable.

## Appendix A

Appendix A shows the floor plan of the three rooms involved in the test procedures of this study. These three rooms are on the same floor and adjoin each other (see Figure A4). The geographical direction is indicated by corresponding compasses.

White areas in the floor plan represent free space in the rooms, while the gray shaded areas indicate furniture such as tables and cabinets. Room 1 contains eight light sources, with light sources 7 and 8 located near the glass window. Room 2 contains ten light sources, with light sources 6 to 10 located towards the windows. Room 3 contains six light sources with no windows.

The green dots represent the test points for static tasks in each room, while the red lines represent the defined paths for walking activities (Figure A1, Figure A2 and Figure A3). The blue dots represent spectrum test points where the local light spectrum was experimentally determined and Table A1 below summarizes the respective heights of the spectrum test points from the floor.

Figure A4 gives the top view of the three rooms with a compass. Further, the definition of the four directions D1 to D4, which were used in the experiments, is also indicated in this figure.

**Table A1:** Height above the floor for the spectrum test points in the three rooms (R1 to R3).

Height (cm)					
Room	S1	S2	S3	S4	S5
R1	118	180	40	180	118
R2	180	142	193	180	140
R3	185	180	140	N/A	N/A

## Appendix B

The temperature of one of the LEDs in room 3 was measured for eight hours after the LED was switched on. The temperature was acquired with a temperature sensor K-type UT TF-K [59] and Figure A5 shows the resulting temperature profile. It can be seen that a constant temperature is established after about one hour and remains stable thereafter.

Thus, the spectral light distributions are compared at three times within this first hour when the LED luminaires are warming up. As can be seen in Figure A6, the spectral distribution remains more or less unchanged despite small changes in illumination intensity. Therefore, the influence on the evaluation algorithm can be neglected, since relative signal features are used in this approach.
